# Association of lower extremity peripheral arterial disease with quantitative muscle features from computed tomography angiography

**DOI:** 10.1186/s13244-024-01663-2

**Published:** 2024-03-27

**Authors:** Ge Hu, Yuexin Chen, Chunlin Mu, Xinyue Zhang, Yun Wang, Yining Wang, Huadan Xue, Daming Zhang, Zhengyu Jin

**Affiliations:** 1 Theranostics and Translational Research Center, National Infrastructures for Translational Medicine, Institute of Clinical Medicine, Peking Union Medical College Hospital, Chinese Academy of Medical Sciences and Peking Union Medical College, Beijing, China; 2 Department of Vascular Surgery, Peking Union Medical College Hospital, Chinese Academy of Medical Sciences and Peking Union Medical College, Beijing, China; 3 Department of Radiology, Peking Union Medical College Hospital, Chinese Academy of Medical Sciences and Peking Union Medical College, Beijing, China; 4 Department of Radiology, Beijing Sixth Hospital, Beijing, China

**Keywords:** Computed tomography angiography, Lower extremity, Muscle feature, Peripheral arterial disease, Vascular stenosis

## Abstract

**Objectives:**

To explore the association between lower extremity muscle features from CTA and peripheral arterial disease (PAD) severity using digital subtraction angiography (DSA) as reference standard.

**Methods:**

Informed consent was waived for this Institutional Review Board approved retrospective study. PAD patients were recruited from July 2016 to September 2020. Two radiologists evaluated PAD severity on DSA and CTA using runoff score. The patients were divided into two groups: mild PAD (DSA score ≤ 7) vs. severe PAD (DSA score > 7). After segmenting lower extremity muscles from CTA, 95 features were extracted for univariable analysis, logistic regression model (LRM) analysis, and sub-dataset analysis (PAD prediction based on only part of the images). AUC of CTA score and LRMs for PAD prediction were calculated. Features were analyzed using Student’s *t* test and chi-squared test. *p* < 0.05 was considered statistically significant.

**Results:**

A total of 56 patients (69 ± 11 years; 38 men) with 56 lower legs were enrolled in this study. The lower leg muscles of mild PAD group (36 patients) showed higher CT values (44.6 vs. 39.5, *p* < 0.001) with smaller dispersion (35.6 vs. 41.0, *p* < 0.001) than the severe group (20 patients). The AUC of CTA score, LRM-I (constructed with muscle features), and LRM-II (constructed with muscle features and CTA score) for PAD severity prediction were 0.81, 0.84, and 0.89, respectively. The highest predictive performance was observed in the image subset of the middle and inferior segments of lower extremity (LRM-I, 0.83; LRM-II, 0.90).

**Conclusions:**

Lower extremity muscle features are associated with PAD severity and can be used for PAD prediction.

**Critical relevance statement:**

Quantitative image features of lower extremity muscles are associated with the degree of lower leg arterial stenosis/occlusion and can be a beneficial supplement to the current imaging methods of vascular stenosis evaluation for the prediction of peripheral arterial disease severity.

**Key points:**

• Compared with severe PAD, lower leg muscles of mild PAD showed higher CT values (39.5 vs. 44.6, *p *< 0.001).

• Models developed with muscle CT features had AUC = 0.89 for predicting PAD.

• PAD severity prediction can be realized through the middle and inferior segment of images (AUC = 0.90).

**Graphical Abstract:**

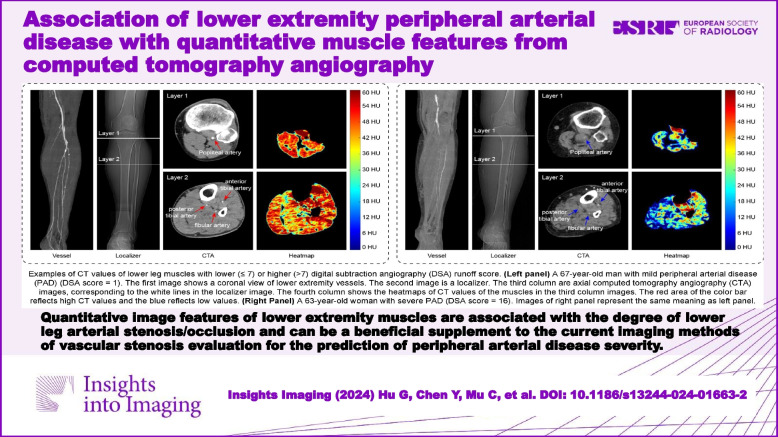

**Supplementary Information:**

The online version contains supplementary material available at 10.1186/s13244-024-01663-2.

## Introduction

Lower extremity peripheral arterial disease (PAD) is an atherosclerotic disease of the lower leg arteries [1–3] that represents a spectrum from asymptomatic stenosis to limb-threatening ischemia [4–6]. More than 200 million people worldwide are living with PAD, and its prevalence continues to rise [4, 7]. Compared with cardiovascular (2.6%) and cerebrovascular (-20.9%) diseases, PAD mortality has increased by 91.9% in the past 20 years [8]. PAD also has a high disability rate, causing a huge economic burden to society and families [9]. Therefore, it is particularly important to accurately assess the PAD severity and implement timely intervention and treatment to prevent patients from developing advanced symptoms that eventually lead to disability [10].

Currently, digital subtraction angiography (DSA) remains the gold standard for determining the degree of vascular stenosis [5]. However, it is now performed less routinely and is replaced by less-invasive imaging techniques, such as computed tomography angiography (CTA) [11–14]. With the advantages of high image quality, short imaging time, and low operator dependency, double lower limb CTA is widely used in the preoperative examination and surgical planning of patients who need revascularization. However, CTA has limitations in the evaluation of vascular stenosis. First, CTA evaluation is a subjective qualitative analysis process, and it may be difficult to distinguish severe stenosis from complete occlusion of lower leg vessels with the naked eye. Second, CTA can hardly display microvessels, so it is sometimes difficult for radiologists to evaluate collateral circulation, which leads to negative arterial morphology results in some symptomatic patients.

Considering the above problems with CTA for vascular evaluation, we tried to find other indicators that could be used as a reference for PAD severity. The clinical symptoms of PAD are mainly caused by muscle ischemia [15]. Ischemia (including microvascular occlusion) induces tissue hypoperfusion, inflammation, and mitochondrial dysfunction leading to muscle atrophy [16]. Thus, limb ischemia is associated with decreased skeletal muscle area, increased muscle fat infiltration, and decreased muscle density [17, 18]; these changes can be evaluated by image features (such as CT value). Increasing evidence suggests that muscle image features can reflect tissue perfusion, assist evaluation of lower leg ischemia, and are independent predictors of cardiovascular events among people with PAD [19–21]. Therefore, we considered the feasibility of circumventing the assessment of vascular stenosis and analyzing CT features of the lower extremity to evaluate muscle ischemia/perfusion and conduct a quantitative auxiliary analysis of PAD severity. The significance of this study is to provide additional reference information for clinical practice to help optimize the diagnostic and therapeutic path for PAD, particularly in patients with discrepancies between imaging signs and clinical symptoms.

In this study, we aimed to investigate the relationship between quantitative lower leg muscle features and PAD severity using DSA as the gold standard, and established logistic regression models (LRM) for PAD severity prediction based on the selected muscle features. Area under the receiver operating characteristic curve (AUC) of CTA score and LRMs was calculated to compare performance.

## Methods

### Patient selection

**Fig. 1:**
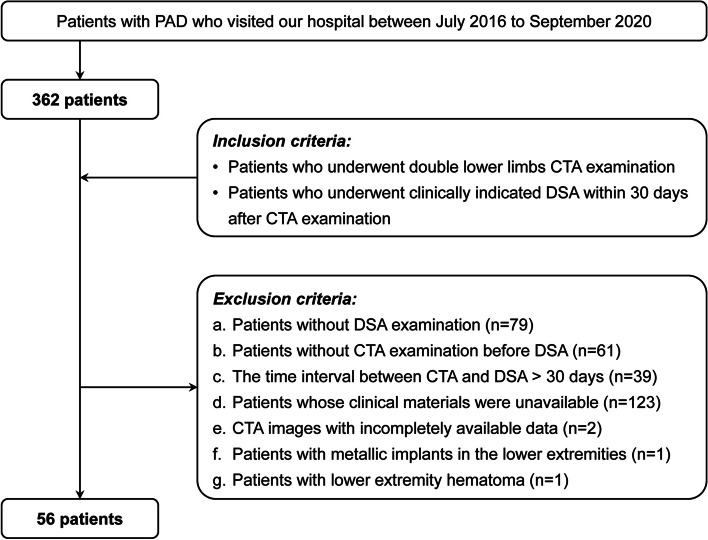
Patient selection flowchart. *PAD *peripheral arterial disease, *CTA *computed tomography angiography, *DSA *digital subtraction angiography

The Institutional Review Board of Peking Union Medical College Hospital approved this retrospective study and waived the requirement for informed consent. We initially enrolled 362 patients with PAD who visited our hospital between July 2016 and September 2020. After reviewing the electronic medical records, 306 patients who did not meet the criteria were excluded (Fig. 1). Ultimately, 56 patients (56 lower limbs) were included in the study. The clinical data, CTA images, and DSA examination results of the enrolled patients were collected for further analysis. Details of patient selection criteria, CT protocol, and DSA protocol are set out in Supplementary Material.

### Image evaluation

The severity and extent of PAD were evaluated using the modified Society for Vascular Surgery runoff score based on DSA and CTA, respectively [5, 6]. The runoff score ranged from 0 to 19, with a higher score indicating more severe disease, and was calculated by assessing the patency and degree of stenosis/occlusion in the lower leg artery segments [22–24]. According to the DSA results, the enrolled patients were divided into two groups: mild PAD (DSA score ≤ 7) and severe PAD (DSA score > 7) [25, 26]. The CTA score was used for comparison with the lower leg muscle features for PAD severity assessment. Details of the runoff score can be found in Supplementary Material.

Both DSA and CTA were analyzed by two vascular imaging radiologists (10-years’ experience; 3-years’ experience), and the consistency of the scores between the two readers were assessed using intraclass correlation coefficient (ICC). Ambiguous results were discussed together to reach a conclusion by consensus. The radiologists first assessed the CTA runoff score and then the DSA runoff score. The washout period between the two readings was more than 3 months and each reading session was completed within 1 week.

### Image segmentation

**Fig. 2:**
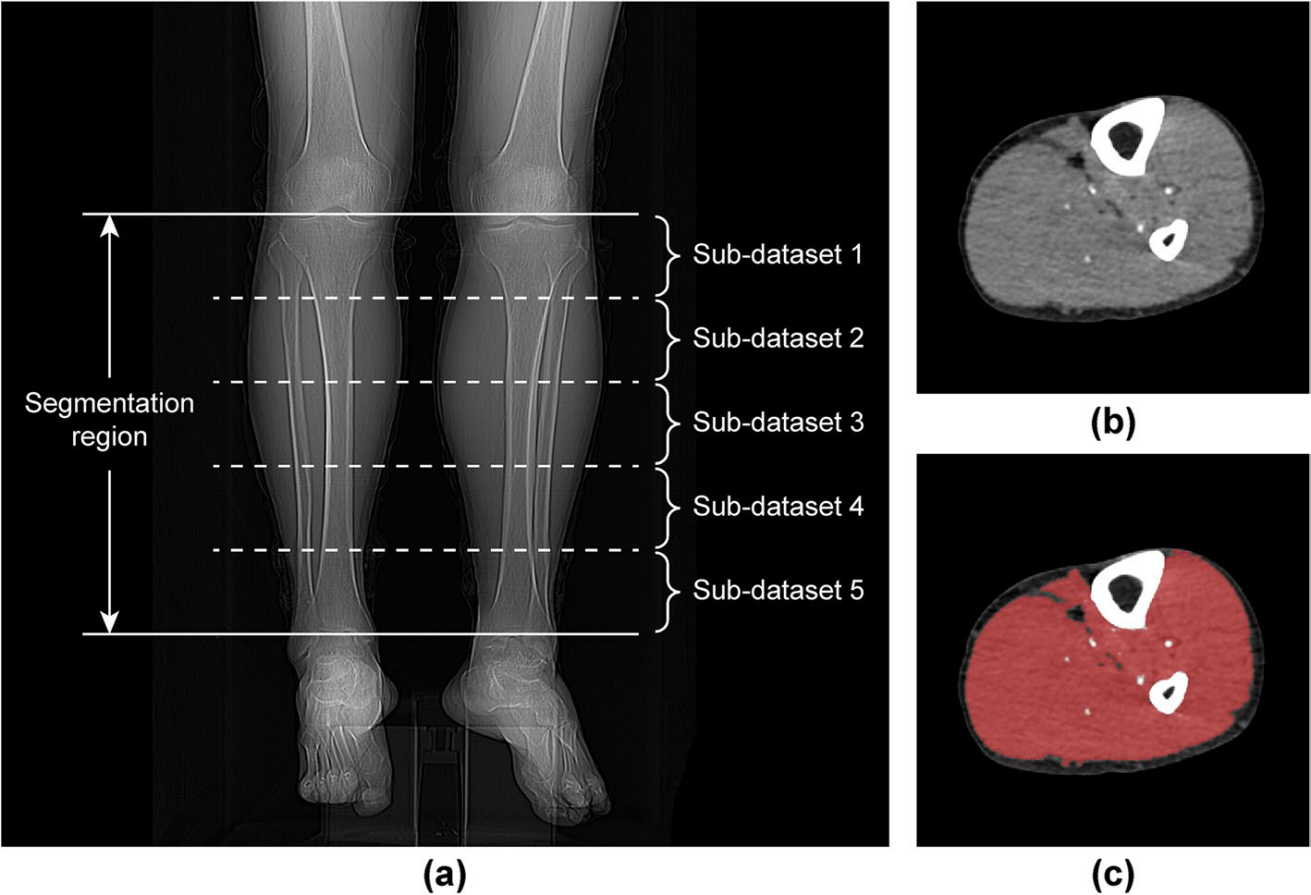
Image segmentation and data division. **a** The segmentation region was from the inferior border of the patella to the superior border of the talus. This part of the lower leg was divided into five equal segments. The computed tomography angiography (CTA) images in each segment constituted an independent dataset, and sub-datasets were constructed (1 to 5 in order from knee to ankle). **b** An original axial CTA image of the lower extremity. **c** The red area represents the segmentation results of the lower leg muscles in image **b**

The lower leg muscle was segmented from the CTA scans using a semi-automatic method based on the threshold technique (Fig. 2). The segmentation region was from the inferior border of the patella to the superior border of the talus, and the CT attenuation threshold was between -10 Hounsfield unit (HU) and 100 HU [20, 27]. After the preliminary separation of muscles and other tissues (fat, bone, and artery), the segmentation results were corrected manually to remove the interference structures (bone marrow and veins), which cannot be segmented correctly by the automatic process only. The above process was realized using MATLAB (version R2020b, MathWorks, Massachusetts) programming.

### Feature extraction

Based on the segmentation results of the lower extremity muscles, 20 histogram features and 75 texture features were extracted from the entire region of interest. Histogram features, such as energy, entropy, coefficient of variation (CV), and interquartile range (IQR), describe the distribution of intensities within the image region [28]. Texture features quantify the relationship between voxels and their surroundings, for both distance and intensity [29]. In total, 95 features were extracted from the interested region of the CTA slices using Python (version 3.7, Python Software Foundation, Delaware) programming. Detailed description of these features is provided in Supplementary Material.

Furthermore, 2 conventional shape features and 14 radiomic shape features were also automatic extracted from the lower limb muscles for further analysis.

### Univariable analysis

The clinical characteristics, histogram features, and texture features were analyzed in this study. *Z*-score normalization was used to standardize the original data to ensure comparability between histogram and texture features. In univariable analysis, continuous variables were analyzed using Student’s *t* test or Mann-Whitney *U* test. Categorical variables were compared using the chi-squared test or Fisher’s exact test. *p* values less than 0.05 were considered to indicate significant differences. Statistical analyses were performed using SPSS (version 26.0, IBM, Armonk). Details of univariable analysis are shown in Supplementary Materials.

### Logistic regression analysis

Considering the potential multicollinearity of the features, least absolute shrinkage and selection operator (LASSO) regression based on ten-fold cross-validation was used to produce more relevant features [30]. The final selected features were used to construct LRM. Coefficients in the logistic regression equation and odds ratios (OR) with 95% confidence intervals (CI) of variables were recorded. The AUC with 95% CI, cutoff value, classification accuracy, sensitivity, and specificity were used to assess the performance of LRM.

In this study, we established two LRMs (LRM-I and LRM-II) for comparison. The input variables of LRM-I were the muscle features selected by LASSO regression. The variables of LRM-II included not only the selected features, but also the CTA scores. Both LRMs were compared with the predictive performance of the CTA score. All the above processes were performed using SPSS. Specific process of logistic regression analysis is provided in Supplementary Materials.

### Sub-dataset analysis

After completing multivariable analysis and establishing LRMs, we further conducted a sub-dataset analysis to verify whether PAD severity prediction can be realized through only a part of the lower leg images. We divided the segmentation region of the lower leg, as previously defined, into five equal segments. The CT images in each segment constituted an independent dataset, and sub-datasets 1–5 were constructed from the knee to the ankle (Fig. 2a). The muscle features (20 histogram features and 75 texture features) extracted from each sub-dataset were inputted into LRM-I and LRM-II, and the PAD predictive performance of the five subsets was evaluated.

## Results

### Patient characteristics

The comparison of this study with our previously published work [20, 31] can be found in Table S1. The reproducibility of image evaluation results between the two radiologists was excellent for the DSA score (ICC, 0.978; 95% CI, 0.961–0.988; *p* < 0.001) and CTA score (ICC, 0.992; 95% CI, 0.990–0.994; *p* < 0.001).

A total of 56 patients (38 men; median age, 69 years (IQR, 64–78 years)) with 56 lower limbs were included in this study. The patients were divided into two groups according to the DSA runoff score. The group with a low DSA score (≤ 7) included 36 patients (26 men; median age, 68 years (IQR, 63–78 years); mean DSA score, 3.3 ± 2.6; mean CTA score, 6.1 ± 4.0), and the group with a high DSA score (> 7) included 20 patients (12 men; median age, 70 years [IQR, 64–79 years]; mean DSA score, 12.3 ± 3.0; mean CTA score, 11.8 ± 5.1).

The clinical characteristics of the enrolled patients are shown in Table 1. The height of the patients was 1.7 m (IQR, 1.6–1.7 m), the weight was 68 ± 12 kg, and the BMI was 25.2 kg/m^2^ (IQR, 22.2–27.4 kg/m^2^). In the study cohort, 15 patients (27%, 15/56) had coronary heart disease, 41 (73%, 41/56) had hypertension, 31 (55%, 31/56) had diabetes, 7 (13%, 7/56) had hyperlipidemia, 31 (55%, 31/56) had a smoking history, and 18 (32%, 18/56) had a history of drinking alcohol. As shown in Table 1, there was no statistical difference in the clinical features between the two groups.

**Table 1:** Demographics and clinical risk factors of the enrolled patients

Characteristics	All *n* = 56	DSA score ≤ 7 *n* = 36	DSA score > 7 *n* = 20	*p* value
Age (years)	69 (64, 78)	68 (63, 78)	70 (64, 79)	0.66
Sex	…	…	…	0.35
Men	38 (68%)	26 (72%)	12 (60%)	…
Women	18 (32%)	10 (28%)	8 (40%)	…
Height (m)	1.7 (1.6, 1.7)	1.7 (1.6, 1.7)	1.6 (1.6, 1.7)	0.69
Weight (kg)	68 ± 12	69 ± 10	65 ± 15	0.32
BMI (kg/m^2^)	25.2 (22.2, 27.4)	25.4 (22.3, 27.0)	24.4 (20.6, 27.5)	0.51
Coronary heart disease	…	…	…	0.82
Yes	15 (27%)	10 (28%)	5 (25%)	…
No	41 (73%)	26 (72%)	15 (75%)	…
Hypertension	…	…	…	0.10
Yes	41 (73%)	29 (81%)	12 (60%)	…
No	15 (27%)	7 (19%)	8 (40%)	…
Diabetes	…	…	…	0.97
Yes	31 (55%)	20 (56%)	11 (55%)	…
No	25 (45%)	16 (44%)	9 (45%)	…
Hyperlipidemia	…	…	…	0.69
Yes	7 (13%)	4 (11%)	3 (15%)	…
No	49 (87%)	32 (89%)	17 (85%)	…
Smoking history	…	…	…	0.09
Yes	31 (55%)	23 (64%)	8 (40%)	…
No	25 (45%)	13 (36%)	12 (60%)	…
Alcohol consumption	…	…	…	0.39
Yes	18 (32%)	13 (36%)	5 (25%)	…
No	38 (68%)	23 (64%)	15 (75%)	…
Fontaine stage^a^	…	…	…	< 0.001
Stage 1	0 (0%)	0 (0%)	0 (0%)	…
Stage 2	39 (70%)	31 (86%)	8 (40%)	…
Stage 3	5 (9%)	1 (3%)	4 (20%)	…
Stage 4	8 (14%)	1 (3%)	7 (35%)	…
Unknown	4 (7%)	3 (8%)	1 (5%)	…

Unless otherwise indicated, continuous variables are presented as mean ± standard deviation or median (interquartile range). Categorical variables are presented as numbers (percentage)

*Abbreviations: DSA* Digital subtraction angiography, *BMI* Body mass index

^a^Fontaine stage: stage 1 indicates numbness and coldness in the lower limbs; stage 2 indicates intermittent claudication; stage 3 indicates ischemic rest pain; and stage 4 indicates necrotic tissue ulceration

### Results of univariable analysis

Table 2 presents the results of the univariate analysis of the 20 histogram features extracted from the lower leg muscles. Seven features, which measured the intensity of CT values from different aspects, showed significant differences between the two groups (DSA score ≤ 7 vs. DSA score > 7): 10th percentile (24.2 vs. 19.0, *p* < 0.001), 90th percentile (64 vs. 61, *p* = 0.006), energy (6.8 × 10^8^ vs. 5.2 × 10^8^, *p* = 0.04), mean (44.6 vs. 39.5, *p* < 0.001), median (45 vs. 39, *p* < 0.001), mode (47 vs. 40, *p* = 0.001), and root mean squared (47.2 vs. 43.1, *p* < 0.001). All these features showed higher values in the mild PAD group.

**Table 2:** Univariable analysis of the histogram features of the lower leg muscles

Features	All *n* = 56	DSA score ≤ 7 *n* = 36	DSA score > 7 *n* = 20	*p* value
10th Percentile	22.4 ± 5.3	24.2 ± 5	19.0 ± 4	< 0.001^*^
90th Percentile	63 (60, 65)	64 (63, 65)	61 (57, 64)	0.006^*^
CV	37.2 (34.4, 41.8)	35.6 (32.8, 38.2)	41.0 (38.9, 45.9)	< 0.001^*^
Energy (10^8^)	6.3 ± 2.7	6.8 ± 2.7	5.2 ± 2.4	0.04^*^
Entropy	1.6 ± 0.1	1.5 ± 0.1	1.6 ± 0.1	0.32
IQR	20 (19, 21)	20 (19, 21)	21 (20, 22)	0.009^*^
Kurtosis	3.8 (3.5, 4.2)	3.8 (3.5, 4.3)	3.6 (3.5, 3.9)	0.20
Maximum	156.6 ± 14.2	157.6 ± 14.3	154.8 ± 13.8	0.50
Mean	44.0 (39.2, 45.8)	44.6 (43.0, 47.0)	39.5 (37.0, 43.5)	< 0.001^*^
MAD	12.6 ± 0.9	12.4 ± 0.9	12.9 ± 0.7	0.04^*^
Median	44 (39, 46)	45 (43, 47)	39 (36, 44)	< 0.001^*^
Minimum	-61.4 ± 16.6	-64.4 ± 16.7	-56.1 ± 15	0.08
Mode	45 (39, 47)	47 (44, 48)	40 (34, 44)	0.001^*^
Range	218.0 ± 24.1	221.9 ± 23.2	210.9 ± 24.2	0.11
RMAD	8.6 ± 0.7	8.5 ± 0.7	8.9 ± 0.6	0.01^*^
RMS	45.8 ± 4.5	47.2 ± 4	43.1 ± 4	< 0.001^*^
Skewness	0.1 (0.0, 0.4)	0.0 (0.0, 0.2)	0.3 (0.1, 0.6)	0.002^*^
SD	16.1 (15.7, 16.9)	16.0 (15.5, 16.4)	16.7 (16.0, 17.1)	0.03^*^
Uniformity	0.4 (0.4, 0.4)	0.4 (0.4, 0.4)	0.4 (0.4, 0.4)	0.26
Variance	260 (247, 286)	256 (241, 270)	280 (255, 294)	0.03^*^

Unless otherwise indicated, data are mean ± standard deviation or median (interquartile range)

*Abbreviations: DSA* Digital subtraction angiography, *CV* Coefficient of variation, *IQR* Interquartile range, *MAD* Mean absolute deviation, *RMAD* Robust mean absolute deviation, *RMS* Root mean squared, *SD* Standard deviation

^*^p values less than 0.05

In addition, there were significant differences between the groups in the following seven features that indicate the dispersion or deviation of CT values (DSA score ≤ 7 vs. DSA score > 7): CV (35.6 vs. 41.0, *p* < 0.001), IQR (20 vs. 21, *p* = 0.009), mean absolute deviation (12.4 vs. 12.9, *p* = 0.04), robust mean absolute deviation (8.5 vs. 8.9, *p* = 0.01), skewness (0.0 vs. 0.3, *p* = 0.002), standard deviation (16.0 vs. 16.7, *p* = 0.03), and variance (256 vs. 280, *p* = 0.03). These features showed higher dispersion in the severe PAD group. For the other six histogram features, no differences were observed between the two groups. Details of the statistical results are shown in Table 2.

**Fig. 3:**
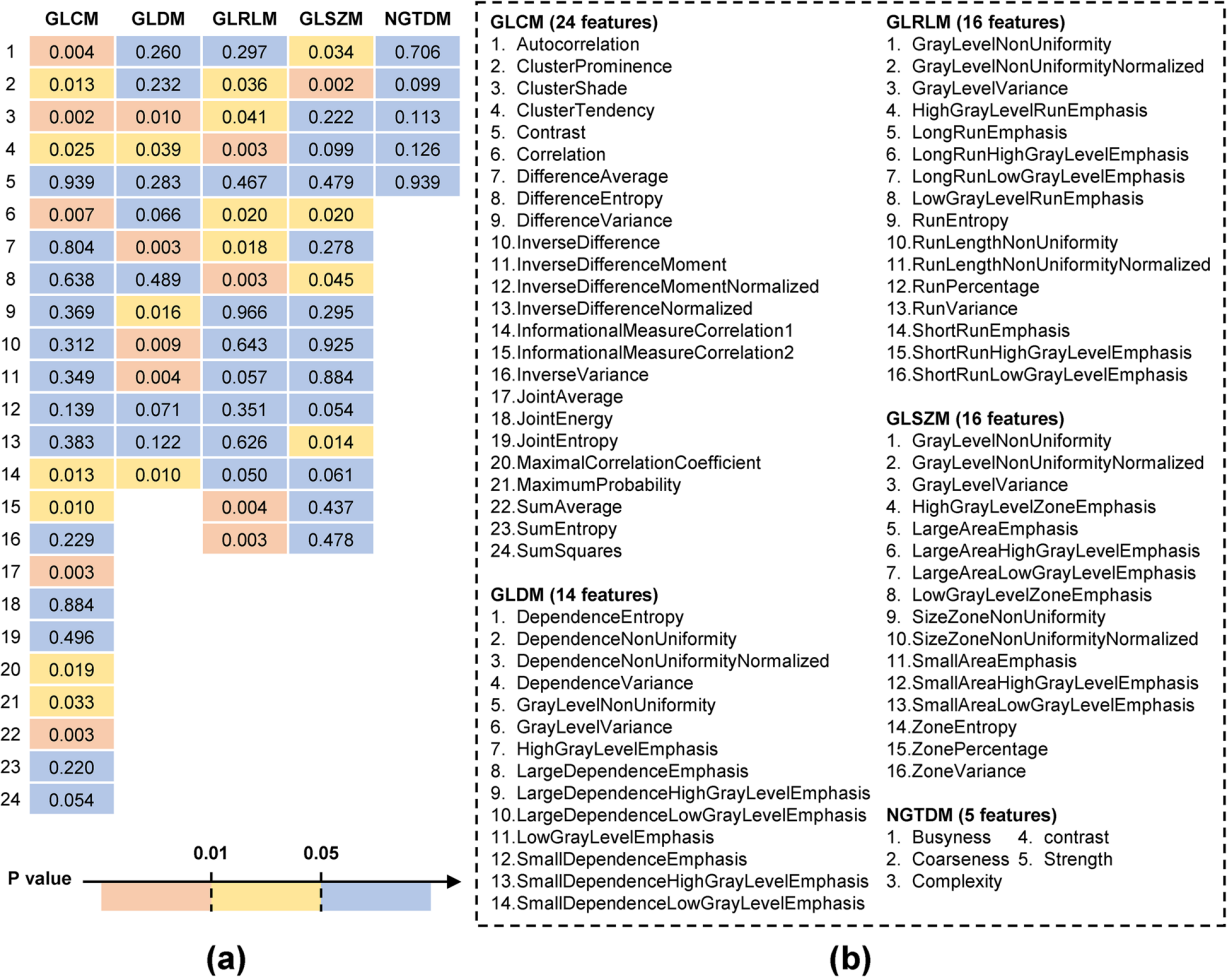
Univariable analysis of texture features of the lower leg muscles. Texture features contain 75 statistics in 5 categories: 24 gray level co-occurrence matrix (GLCM) features, 14 gray level dependence matrix (GLDM) features, 16 gray level run length matrix (GLRLM) features, 16 gray level size zone matrix (GLSZM) features, and 5 neighboring gray tone difference matrix (NGTDM) features. **a** The *p* values of the statistical analysis of the 75 texture features. The values in the red, yellow, and blue areas represent *p* < 0.01, 0.01 < *p* < 0.05, and *p* > 0.05, respectively. **b** Feature names corresponding to (**a**). The numbers before the feature names in **b** corresponding to the leftmost line number in **a**

Figure 3 shows the analysis results of the 75 texture features. Figure 3a presents the *p* values of these features. The values in the red, yellow, and blue areas represent *p* < 0.01, 0.01 < *p* < 0.05, and *p* > 0.05, respectively. Figure 3b was the feature names corresponding to Fig. 3a. As shown in Fig. 3, there were significant differences between the two groups in 31 texture features. Results of the shape features can be found in Table S2 and Supplementary Materials. Compared to the severe PAD group, the mild PAD group had larger muscle volume and average area (volume (cm^3^), 1084 vs. 876, *p* = 0.01; area (cm^2^), 32 vs. 25, *p* = 0.04).

### Results of logistic regression analysis

**Fig. 4:**
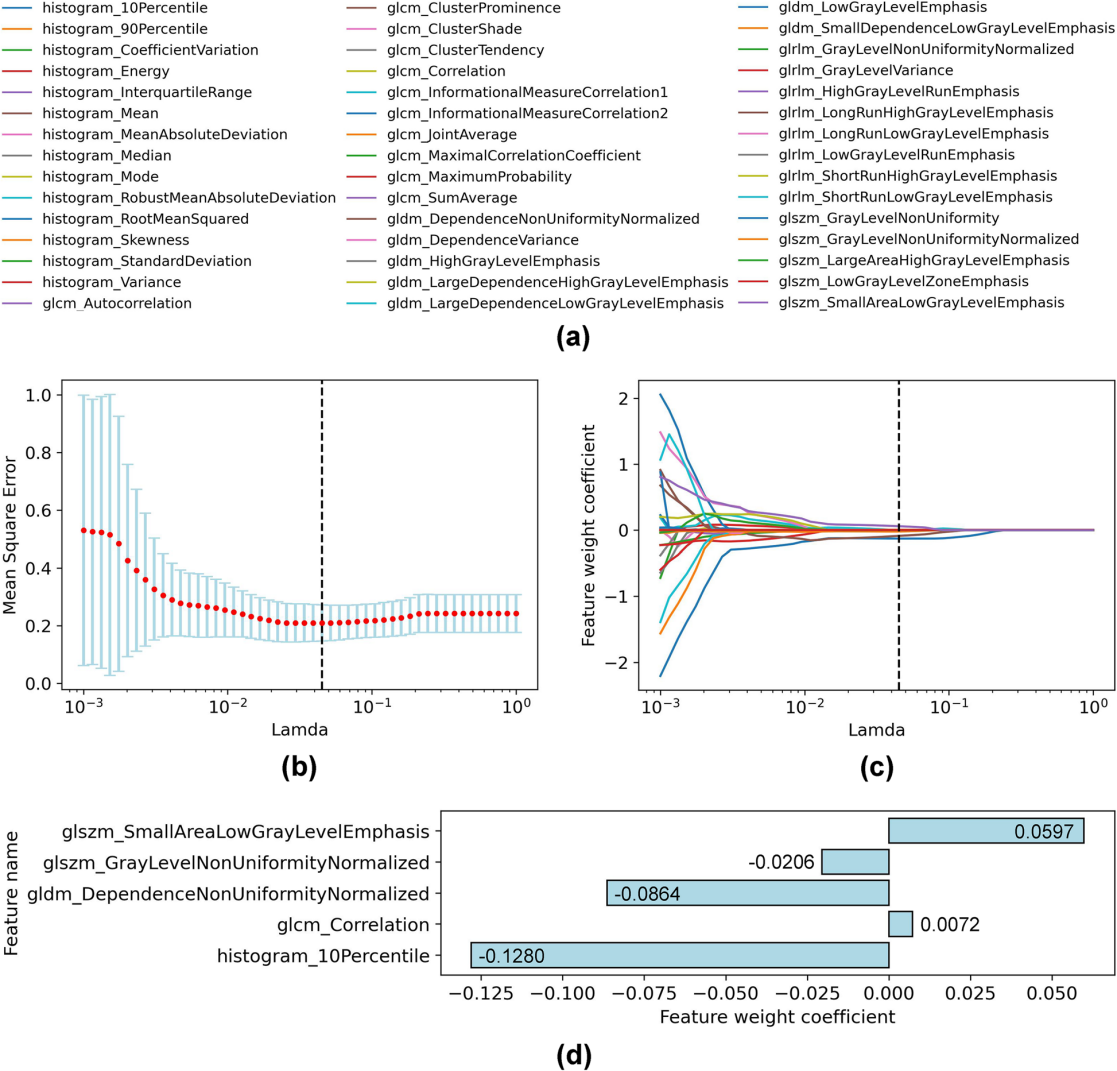
Feature selection based on least absolute shrinkage and selection operator (LASSO) regression. **a** Forty-five features (14 histogram features and 31 texture features) with *p* < 0.05 selected by univariable analysis. **b** The trend graph of the mean square error (MSE) with different *λ* (Lamda) during cross-validation. *λ* is an important parameter of LASSO regression that is usually adjusted by cross-validation to find the optimal value. The red dots represent the average values of the MSE. The blue error bars represent the standard deviation of the MSE. The black dotted line indicates the best value of *λ*. **c** The convergence graph of the weight coefficients of the features under different *λ* values. Each convergence line corresponds to a feature, and the color of the line matches the color before the feature name in **a**. As shown in **b** and **c**, the MSE is minimized (0.21 ± 0.07) at *λ* = 0.044 (the black dotted line), where five representative features were finally identified (weight coefficient ≠ 0). **d** Feature names and weight coefficients of the five selected features. *GLCM *gray level co-occurrence matrix, *GLDM *gray level dependence matrix, *GLRLM *gray level run length matrix, *GLSZM *gray level size zone matrix

Considering the collinearity between muscle features, we further selected 45 features (14 histogram features and 31 texture features) with *p* < 0.05 using LASSO regression. As shown in Fig. 4, five representative image features were finally identified. Figure 4a–c describes the process of feature selection, and Fig. 4d shows the feature names and weight coefficients of the final selected features.

Table 3 presents the multivariable analysis results of the two LRMs (LRM-I and LRM-II). As shown in Table 3, histogram_10Percentile (OR, 0.28; 95% CI, 0.13–0.61; *p* = 0.001) and gldm_DependenceNonUniformityNormalized (OR, 0.51; 95% CI, 0.27–0.98; *p* = 0.04) retained significant difference in LRM-I. CTA runoff score (OR, 3.27; 95% CI, 1.42–7.53; *p* = 0.006), histogram_10Percentile (OR, 0.33; 95% CI, 0.14–0.77; *p* = 0.01), and gldm_DependenceNonUniformityNormalized (OR, 0.51; 95% CI, 0.25–1.03; *p* = 0.06) were eventually selected in LRM-II.

**Table 3:** Multivariable analysis results of the two logistic regression models for peripheral arterial disease prediction

Variables in logistic regression model	Coefficient^a^	OR (95% CI)	*p* value
***LRM-I***			
histogram_10Percentile	-1.29	0.28 (0.13, 0.61)	0.001
glcm_Correlation	NR	NR	NR
gldm_DependenceNonUniformityNormalized	-0.67	0.51 (0.27, 0.98)	0.04
glszm_GrayLevelNonUniformityNormalized	NR	NR	NR
glszm_SmallAreaLowGrayLevelEmphasis	NR	NR	NR
Constant^b^	-0.85	0.43 (NR, NR)	0.02
***LRM-II***			
CTA runoff score	1.18	3.27 (1.42, 7.53)	0.006
histogram_10Percentile	-1.11	0.33 (0.14, 0.77)	0.01
glcm_Correlation	NR	NR	NR
gldm_DependenceNonUniformityNormalized	-0.68	0.51 (0.25, 1.03)	0.06
glszm_GrayLevelNonUniformityNormalized	NR	NR	NR
glszm_SmallAreaLowGrayLevelEmphasis	NR	NR	NR
Constant^b^	-0.90	0.41 (NR, NR)	0.03

*Abbreviations: OR* Odds ratio, *CI* Confidence interval, *LRM* Logistic regression model, *GLCM* Gray level co-occurrence matrix, *GLDM* Gray level dependence matrix, *GLSZM* Gray level size zone matrix, *CTA* Computed tomography angiography, *NR* Not reported

^a^Coefficient of variables in logistic regression equation

^b^Intercept in logistic regression equation

According to the coefficients of variables and the constant in Table 3, we establish logistic regression equations of the two models: LRM-I, $Logit\left(P\right)=-1.29\times {\text{Feature}}1-0.67\times {\text{Feature}}2-0.85$; LRM-II, $Logit\left(P\right)=1.18\times \mathrm{CTA score}-1.11\times {\text{Feature}}1-0.68\times {\text{Feature}}2-0.90$. In the two equations, Logit (·) represents logit transformation, P represents the output probability of the LRMs, Feature1 represents the value of histogram_10Percentile, and Feature2 represents the value of gldm_DependenceNonUniformityNormalized.

Table 4 shows the performance of the CTA runoff score and the LRMs constructed with lower leg muscle features. Both LRMs were significant (Omnibus test, *p* < 0.001) with a high goodness of fit (Hosmer–Lemeshow test, *p* > 0.05). Compared to the CTA score (AUC, 0.81; sensitivity, 75%; specificity, 75%; accuracy, 75%), LRM-I achieved a better predictive performance (AUC, 0.84; sensitivity, 80%; specificity, 81%; accuracy, 80%). LRM-II achieved the best results (AUC, 0.89; sensitivity, 80%; specificity, 83%; accuracy, 82%).

**Table 4:** Comparison of CTA score and logistic regression models for PAD prediction

Evaluation index	CTA runoff score	LRM-I	LRM-II
Omnibus test^†^	…	< 0.001	< 0.001
Hosmer and Lemeshow test^‡^	…	0.19	0.90
AUC (95% CI)	0.81 (0.69, 0.92)	0.84 (0.73, 0.94)	0.89 (0.80, 0.97)
*p* value of AUC	< 0.001	< 0.001	< 0.001
Sensitivity	75 (15/20)	80 (16/20)	80 (16/20)
Specificity	75 (27/36)	81 (29/36)	83 (30/36)
Classification accuracy	75 (42/56)	80 (45/56)	82 (46/56)
Cutoff value^a^	9.50	0.35	0.43

*Abbreviations: CTA* Computed tomography angiography, *PAD* Peripheral arterial disease, *LRM* Logistic regression model, *AUC* Area under curve, *CI* Confidence interval

^†^*p* value of Omnibus test

^‡^*p* value of Hosmer and Lemeshow test

^a^Selection criterion of the cutoff value: the highest value of Youden index (sensitivity + specificity − 1)

**Fig. 5:**
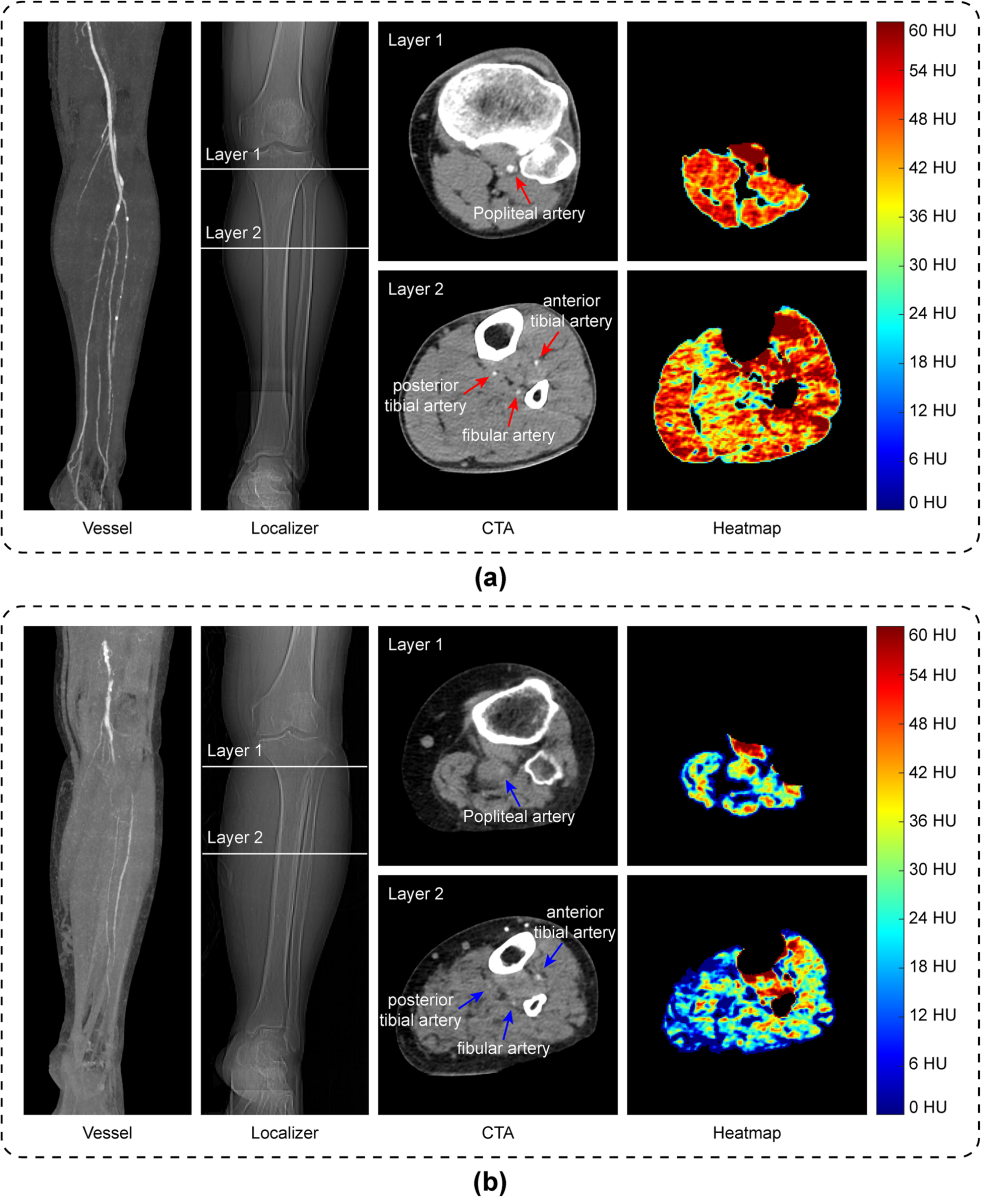
Examples of CT values of lower leg muscles with lower (≤ 7) or higher (> 7) digital subtraction angiography (DSA) runoff score. **a** A 67-year-old man with mild peripheral arterial disease (PAD) (DSA score = 1). The first image shows a coronal view of lower extremity vessels. The second image is a localizer. The third column are axial computed tomography angiography (CTA) images, corresponding to the white lines in the localizer image. The fourth column shows the heatmaps of CT values of the muscles in the third column images. The red area of the color bar reflects high CT values and the blue reflects low values. **b** A 63-year-old woman with severe PAD (DSA score = 16). Images of **b** represent the same meaning as **a**

Figure 5 shows two examples of CT values of lower leg muscles with lower (≤ 7) or higher (> 7) DSA runoff score. Comparing the two sets of heatmaps, the lower leg muscles of patient with mild PAD is shown as red regions with higher CT values. And the patient with severe PAD is shown as blue regions with lower CT values.

### Results of sub-dataset analysis

To validate whether the severity of PAD can be predicted using only partial lower extremity images, we extracted muscle features from sub-datasets 1 to 5 and input them into the logistic regression equations of LRM-I and LRM-II to evaluate the predictive performance of each subset. The results (Table 5) showed that the AUC of LRM-I on sub-datasets 1 to 5 were 0.79, 0.79, 0.82, 0.83, and 0.78, respectively. The AUC of LRM-II on the subsets were 0.86, 0.86, 0.88, 0.90, and 0.86, respectively. The trend of the AUC values for the five subsets was consistent for both LRMs, and the largest AUC were obtained in sub-dataset 4 (LRM-I, 0.83; LRM-II, 0.90). Details of the other evaluation indices can be found in Table 5.

**Table 5:** Performance of logistic regression models on the five sub-datasets

Sub-dataset^a^	AUC (95% CI)	*p* value	Sensitivity	Specificity	Accuracy	Cut-point
***Sub-dataset 1***						
LRM-I	0.79 (0.68, 0.91)	< 0.001	60 (12/20)	75 (27/36)	70 (39/56)	0.35
LRM-II	0.86 (0.76, 0.96)	< 0.001	70 (14/20)	81 (29/36)	77 (43/56)	0.43
***Sub-dataset 2***						
LRM-I	0.79 (0.66, 0.91)	< 0.001	70 (14/20)	64 (23/36)	66 (37/56)	0.35
LRM-II	0.86 (0.76, 0.96)	< 0.001	75 (15/20)	83 (30/36)	80 (45/56)	0.43
***Sub-dataset 3***						
LRM-I	0.82 (0.70, 0.93)	< 0.001	80 (16/20)	78 (28/36)	79 (44/56)	0.35
LRM-II	0.88 (0.79, 0.97)	< 0.001	75 (15/20)	81 (29/36)	79 (44/56)	0.43
***Sub-dataset 4***						
LRM-I	0.83 (0.72, 0.94)	< 0.001	85 (17/20)	69 (25/36)	75 (42/56)	0.35
LRM-II	0.90 (0.83, 0.98)	< 0.001	70 (14/20)	81 (29/36)	77 (43/56)	0.43
***Sub-dataset 5***						
LRM-I	0.78 (0.67, 0.90)	< 0.001	70 (14/20)	72 (26/36)	71 (40/56)	0.35
LRM-II	0.86 (0.77, 0.96)	< 0.001	60 (12/20)	86 (31/36)	77 (43/56)	0.43

*Abbreviations: AUC* Area under curve, *CI* Confidence interval, *LRM* Logistic regression model

^a^The segmentation region (from the inferior border of the patella to the superior border of the talus) of the lower leg muscles was divided into five equal segments. The CT images in each segment constituted an independent dataset, and sub-datasets 1–5 were constructed from the knee to the ankle

## Discussion

In this study, we collected CTA images and clinical data of patients with PAD and divided them into a mild PAD group (DSA runoff score ≤ 7) and a severe PAD group (DSA runoff score > 7). After segmenting the lower limb muscles and extracting CT features, we analyzed the relationship between the muscle features and severity of PAD. The results showed that there was a significant difference in the histogram features of the lower extremity muscles between the two groups, and the mild group had higher CT values (mean, 44.6 HU vs. 39.5 HU, *p* < 0.001) with smaller dispersion (CV, 35.6 vs. 41.0, *p* < 0.001) than the severe group. Since the CT value of blood is usually higher than that of soft tissues, it is reasonable that patients in the mild PAD group had better blood flow and muscle perfusion in the lower extremities and therefore obtained higher feature values.

In addition, we established two LRMs based on the muscle features, without (LRM-I) or with (LRM-II) CTA runoff score. Compared with the independent predictive performance of the CTA score, LRM-I, the model containing only muscle features, obtained more accurate results (CTA vs. LRM-I, 0.81 vs. 0.84). This suggests that CTA features of the lower leg muscles can assist in the assessment of PAD severity and hold promise as a useful complement to CTA. Meanwhile, LRM-II, the model containing both imaging features and CTA scores, showed the highest AUC value (0.89), which indicates that richer features incorporated in the model are associated with improved predictive performance.

Due to the relatively long lower extremities, even with a thickness of 5 mm, each patient’s lower leg, from the patella to the talus, still covers 60–70 CTA slices. In addition, because of limitations in the generalization of current automatic muscle segmentation techniques, it is somewhat unrealistic to ask doctors to segment the muscles of the entire lower limb. Therefore, we tested the previously developed LRMs on the five sub-datasets that were divided from the original dataset to verify whether PAD severity can be predicted from only a part of the lower leg images. The results showed that even a segment of the lower leg images still had the ability to predict PAD severity. And the highest performance was found in sub-dataset four (images of middle and inferior segments of the lower extremity) along the knee-to-ankle direction. This finding is reasonable because the clinical symptoms of muscle ischemia caused by upper vascular stenosis were usually more obvious distally.

In addition to CTA, imaging technologies currently used in the clinical scenario of PAD include duplex ultrasound imaging (DUS) and magnetic resonance angiography (MRA) [2, 5]. DUS can identify the anatomical location of the disease and determine the severity of focal stenosis. However, DUS is operator-dependent and sometimes cannot be used in overweight patients. MRA is also useful for assessing PAD anatomy and the presence of stenosis, but it may be inaccurate in arteries treated with metal stents. Furthermore, there are many new methods for PAD severity assessment, such as deep learning-based classification models [32], non-contrast QISS-MRA [33, 34], near infrared spectroscopy [35], and some dynamic imaging technology [31, 36].

For muscle perfusion, several previous studies have confirmed its relationship with muscle ischemia using MR arterial spin labeling [19, 37, 38]. The results showed that blood flow gradually decreased with increasing severity of limb ischemia, whereas model arterial resistance progressively increased. In addition, low leg muscle density on CT was proved to be associated with increased risk of lower limb events (rate ratio 1.41) and was a strong, independent predictor of major cardiovascular events in people with PAD [21]. Our previous published work has also validated the association between lower leg enhancement on dynamic CTA and PAD severity [20]. Compared with previous studies, this study further developed a prediction model for PAD severity based on standard CTA features and tested its adaptability on a subset of lower limb images, thus further validating the feasibility, accuracy, and convenience of lower leg muscle features as an indicator of PAD assessment.

In clinical practice, discrepancies between imaging findings and clinical symptoms are sometimes observed. For example, mild claudication with severe vessel stenosis, or ischemic rest pain but negative lower extremity arterial abnormalities. Whether these patients should receive timely intervention and the therapeutic effect that can be achieved after treatment are usually confusing to clinicians. Our research demonstrate that quantitative muscle features are associated with PAD severity and have the potential to be an indicator for PAD prediction. These features provide clinicians with additional perfusion information, which usually reflects the true blood supply to the lower limb. However, the morphology of the microvasculature is difficult to be assessed using conventional imaging examinations such as CTA.

Our study has several limitations. (a) This was a retrospective study which enrolled only 56 patients as most patients were excluded because of missing data. Therefore, this study lacks a re-test dataset and the findings need to be validated by prospective studies based on a larger sample. (b) Limited by existing segmentation techniques, we used a semi-automatic method for lower extremity muscle segmentation to ensure accurate feature extraction. Although we verified that a segment of the entire image has predictive ability, this approach limits the generalization of this method. (c) The CT attenuation-based values of lower limb muscle features analyzed in this study were derived from the single-phase CTA images, which is probably too prone for artifacts and incorrect phase imaging. In the future, we will further explore the value of lower extremity muscle features in the prediction of PAD severity in conjunction with other imaging techniques, such as perfusion imaging, dual-energy imaging and photon-counting imaging. (d) Collateral vessels play an important role in the vascular assessment of PAD patients. However, due to the lack of collateral vessel evaluation in the modified SVS runoff score and the limited demonstration of collateral circulation on CTA, it was difficult to assign an appropriate and reasonable weight (relative to other lower extremity vessels) to collateral vessels and include it in the current scoring system. (e) Although we used a semi-automatic segmentation process, combining the threshold algorithm and manual correction, to remove fat from the CT images as much as possible, we were unable to completely exclude the influence of adipose tissue (especially intramuscular fat) due to differences in patients and imaging equipment.

In conclusion, CTA features of lower extremity muscle are associated with PAD severity and can be used for PAD prediction, and to compensate for the limitations of vascular stenosis assessment from the perspective of muscle ischemia evaluation. In the future, we will further explore the feasibility of PAD severity prediction based on the radiomic features of non-contrast CT images.

### Supplementary Information

**Supplementary Material 1.**

## Data Availability

The datasets used and/or analyzed during the current study are available from the corresponding author on reasonable request.

## Abbreviations

AUC: Area under the curve
CI: Confidence interval
CTA: Computed tomography angiography
CV: Coefficient of variation
DSA: Digital subtraction angiography
HU: Hounsfield unit
ICC: Intraclass correlation efficient
IQR: Interquartile range
LASSO: Least absolute shrinkage and selection operator
LRM: Logistic regression model
OR: Odds ratio
PAD: Peripheral arterial disease
