# Supplementary material for: Association of lower extremity peripheral arterial disease with quantitative muscle features from computed tomography angiography
Source: Insights Imaging. 2024 Mar 27;15:95. doi: 10.1186/s13244-024-01663-2 (PMC10973322; doi:10.1186/s13244-024-01663-2)
Supplement: Supplementary file 1 — Supplementary Material 1. [file 13244_2024_1663_MOESM1_ESM.pdf]

Association of lower extremity peripheral arterial disease with quantitative muscle features from computed tomography angiography

ELECTRONIC SUPPLEMENTARY MATERIAL

Patient Selection Criteria

The inclusion criteria were as follows: (a) patients who underwent double lower limbs CTA examination; and (b) patients who underwent clinically indicated DSA within 30 days after CTA.

The exclusion criteria were as follows: (a) patients without DSA examination (n=79); (b) patients without CTA examination before DSA (n=61); (c) the time interval between CTA and DSA > 30 days (n=39); (d) patients whose clinical materials were unavailable (n=123); (e) CTA images with incompletely available data (n=2); (f) patients with metallic implants in the lower extremities (n=1); and (g) patients with lower extremity hematoma (n=1).

CTA Protocol

All scans were performed on a dual-layer spectral-detector CT scanner (IQon Spectral CT, Philips Healthcare, Netherlands) and conducted in the cranio-caudal direction. The acquired scan length was defined from the distal abdominal aorta to the toes. The arterial enhancement was achieved with 90 ml of iodinated contrast agent (370 mgI/ml iopromide, Ultravist, Bayer Healthcare, Germany) administered intravenously at a rate of 4 ml/s. A bolus tracking technique was used to trigger the scanning. The trigger level was the distal abdominal aorta and the trigger threshold was 150 Housfield unit (HU). All acquisitions were reconstructed with the thickness of 1 mm (for image evaluation) and 5 mm (for image segmentation), and a soft convolution kernel (B) for all acquisition phases. The field of view was set to 408 mm with a pixel matrix of 512×512. All computed tomography angiography (CTA) images are in accord with the Digital Imaging and Communications in Medicine (DICOM) standard.

DSA Protocol

Digital subtraction angiography (DSA) was performed on an angiographic system (Axiom Artis, Siemens Healthcare, Germany). The pelvic and lower extremity arteries of the symptomatic leg were examined with 5 ml iodinated contrast medium (320 mgI/ml iodixanol, Visipaque, GE Healthcare, USA) per segment (aorta-iliac, femoral-popliteal and below the knees) using the stepping table DSA technique in a posteroanterior projection. If the stenosis could not be assessed from the posteroanterior projection, additional left and right anterior oblique projections were obtained.

Modified SVS Runoff Score

As anatomical features affect endovascular interventions, the anatomical/angiographic disease description in patients with peripheral arterial disease (PAD) requires a scoring system that is sensitive to differences in arterial disease. In this study, the severity and extent of PAD is assessed using the modified SVS runoff score, which is one of the scoring systems recommended by the Society for Vascular Surgery in its published reporting standards [1,2] and provides a comprehensive assessment of the anatomic characteristics of the peripheral arteries. This scoring system has been widely used in previous studies of lower limb vascular disease [3-5].

The score ranges from 0 to 19, with a higher score indicating more severe disease, and is calculated by assessing the patency and degree of stenosis/occlusion in the lower leg artery segments (popliteal artery, anterior tibial artery, posterior tibial artery, and fibular artery). Stenoses or occlusion were rated by estimating the percentage of lumen narrowing compared to the adjacent non-stenotic lumen. A score of 0 is assigned for vessels with <20% stenosis, 1 for 21% to 49% stenosis, 2 for 50% to 99% stenosis, 2.5 for a vascular occlusion less than half its length, and 3 for an occlusion greater than half the vessel length. The score for the popliteal artery is multiplied by 3 and 1 is added to the sum of all 4 vessel scores together, giving a range of possible popliteal artery scores from 1 to 10.

Histogram Features

Histogram features, which are also called first-order features, describe the intensity and distribution of CT attenuation values within the interested region (the lower leg muscles) defined by the mask through commonly used and basic metrics, including the following 20 statistics:

1. 10th Percentile: the 10th percentile CT value within the region of interest (ROI).
2. 90th Percentile: the 90th percentile CT value within the ROI.
3. Coefficient of variation (CV): CV also known as relative standard deviation, is a standardized measure of the dispersion of a probability distribution or frequency distribution of CT values.
4. Energy: energy is a measure of the magnitude of voxel values in a CT image. A larger value implies a greater sum of the squares of these values.
5. Entropy: entropy specifies the uncertainty/randomness in the CT values. It measures the average amount of information required to encode the CT values.
6. Interquartile Range (IQR): the CT values between the 25th and 75th percentile.
7. Kurtosis: kurtosis is a measure of the peakedness of the distribution of CT attenuations in the ROI. A higher kurtosis implies that the mass of the distribution is concentrated towards the tail(s) rather than towards the mean. A lower kurtosis implies the reverse: that the mass of the distribution is concentrated towards a spike near the mean value.
8. Maximum: the maximum CT value within the ROI.
9. Mean: the average CT value within the ROI.

10. Mean absolute deviation (MAD): MAD is the mean distance of all CT values from the mean value of the CT image array.
11. Median: the median CT value within the ROI.
12. Minimum: the minimum CT value within the ROI.
13. Mode: the mode CT value within the ROI.
14. Range: the range of CT values in the ROI.
15. Robust mean absolute deviation (RMAD): RMAD is the mean distance of all CT values from the mean value calculated on the subset of image array with CT values in between, or equal to the 10th and 90th percentile.
16. Root mean squared (RMS): RMS is the square-root of the mean of all the squared CT attenuations. It is another measure of the magnitude of the image values.
17. Skewness: skewness measures the asymmetry of the distribution of CT attenuations about the mean value. Depending on where the tail is elongated and the mass of the distribution is concentrated, this value can be positive or negative.
18. Standard Deviation (SD): SD measures the amount of variation or dispersion from the mean value.
19. Uniformity: uniformity is a measure of the sum of the squares of each CT attenuation. This is a measure of the homogeneity of the image array, where a greater uniformity implies a greater homogeneity or a smaller range of discrete CT values.
20. Variance: Variance is the mean of the squared distances of each CT attenuation from the mean value. This is a measure of the spread of the distribution about the mean.

Texture Features

Texture features quantify the relationship between voxels and their surroundings of both distance and intensity, containing 75 statistics in the following 5 categories: 24 gray level co-occurrence matrix (GLCM) features, 14 gray level dependence matrix (GLDM) features, 16 gray level run length matrix (GLRLM) features, 16 gray level size zone matrix (GLSZM) features, and 5 neighboring gray tone difference matrix (NGTDM) features.

1. GLCM describes the second-order joint probability function of an image region constrained by the matrix that computes how often pairs of pixels with a specific value and offset occur in the image.
2. GLDM quantifies gray level dependencies in an image. A gray level dependency is defined as the number of connected voxels within distance δ that are dependent on the center voxel.
3. GLRLM quantifies gray level runs, which are defined as the length in number of pixels, of consecutive pixels that have the same CT value.
4. GLSZM quantifies CT attenuation zones in an image. A CT attenuation zone is defined as the number of connected voxels that share the same gray level intensity. A voxel is considered connected if the distance is 1 according to the infinity norm.
5. NGTDM quantifies the difference between a CT value and the average CT value of its neighbors within distance δ.

Univariable analysis

To ensure the comparability between histogram features and texture features, Z-score normalization was used to standardize the original data before univariable analysis. This method normalizes the data according to the mean and standard deviation of the original data. Through Z-score standardization, the data of different orders of magnitude are uniformly transformed into the same one, which is measured by the calculated Z-score value to ensure comparability between the data.

In univariable analysis, the Shapiro-Wilk test was used to assess the normality of distribution. Continuous variables of normal distribution were analyzed using Student's t-test and expressed as mean ± standard deviation (SD). Continuous variables of abnormal distribution were analyzed using Mann-Whitney U test and expressed as median with interquartile range (IQR). Categorical variables were compared using the Chi square test or Fisher exact test and reported as numbers with percentage. P>0.05 on Shapiro-Wilk test represent normal distribution. For other tests, P<0.05 were considered to indicate significant differences.

LASSO Regression

Least absolute shrinkage and selection operator (LASSO) regression is a shrinkage algorithm that allows active selection from a set of variables with a large amount of data and potential multicollinearity to produce more relevant and interpretable features. The important parameter λ with the lowest mean square error is selected by ten-fold cross-validation. LASSO regression was realized through Python programming.

In ten-fold cross-validation, the original training set is randomly partitioned into ten equal-sized subsamples. Of the ten subsamples, a single subsample is retained as the validation data for testing the model, and the remaining nine subsamples are used as new training data. The cross-validation process is then repeated ten times, with each of the ten subsamples used exactly once as the validation data. The ten results can then be averaged to produce a single estimation.

Logistic regression analysis

In logistic regression analysis, the variable selection method was forward stepwise regression based on maximum likelihood estimation. The overall evaluation of logistic regression model (LRM) was assessed by Omnibus test, and P values less than 0.05 of Omnibus test represent statistically significant. The goodness-of-fit of LRM was analyzed by Hosmer and Lemeshow test, and P values more than 0.05 of this test represent high goodness-of-fit. Area under the curve (AUC) with 95% CI, cutoff value, classification accuracy, sensitivity, and specificity were used to assess the performance of LRM. The selection criteria of the cut-point were based on the maximum of Youden index (sensitivity+specificity-1).

Results of shape features

Table.S2 shows the univariable analysis results of the shape features of the lower leg muscles. The results showed that the muscle volume and average area of the mild PAD patients were significantly larger than those of the severe PAD patients (volume [cm^3], 1084 vs. 876, P=0.01; area [cm^2], 32 vs. 25, P=0.04), which was consistent with our expectation that severe PAD would lead to muscle atrophy.

In addition, the severe group had a larger muscle surface area and lower sphericity than the mild PAD group (surface area, 67341 vs. 82001, P=0.03; sphericity, 0.32 vs. 0.26, P<0.001). As the threshold-based semi-automatic segmentation process used in this study evaluates each image pixel to exclude non-muscle image points (such as adipose tissue), if there is severe fat infiltration in the muscles, the segmented results of the muscles will be sparse and porous, ultimately increasing the surface area, and decreasing the sphericity. Therefore, the differences in these two features between the PAD groups confirm the presence of more severe fat infiltration in the muscles of the severe PAD group.

SUPPLEMENTARY TABLES

Table S1 Comparison of this study with our previously published work

Comparison	This study	Our previous study 1†	Our previous study 2‡
Objectives	To investigate the association between lower leg muscle features measured from standard CTA (s-CTA) and PAD severity	To explore the relationship between lower leg muscle enhancement measured from dynamic CTA (dyn-CTA) and PAD severity	To evaluate whether time maximum intensity projection CTA (t-MIP CTA) postprocessed from dyn-CTA could be used for PAD diagnosis
Population	PAD patients recruited between July 2016 and September 2020	PAD patients recruited between November 2015 and March 2016	PAD patients recruited between November 2015 and March 2016
Equipment	IQon Spectral CT, Philips Healthcare, Netherlands	Somatom Definition Force, Siemens Healthcare, Germany	Somatom Definition Force, Siemens Healthcare, Germany
Subjective evaluation	The SVS runoff score of DSA and s-CTA	The SVS runoff score of dyn-CTA	The SVS runoff score of s-CTA and t-MIP CTA
Quantitative analysis	① Muscle histogram features (first-order) of s-CTA	① Muscle kinetic parameters of average time attenuation curves of dyn-CTA	Vascular CT attenuation of the best enhancement phase of dyn-CTA and t-MIP CTA

	② Muscle texture features (higher-order) of s-CTA	② Muscle histogram of the first enhanced phase of dyn-CTA	

Abbreviations: CTA = computed tomography angiography. PAD = peripheral arterial disease. SVS = Society for Vascular Surgery. DSA = digital subtraction angiography.

† Previous study 1 was published in J Comput Assist Tomogr 2020;44(1):20-25

‡ Previous study 2 was published in BMC Med Imaging 2021;21(1):7

Table S2 Univariable analysis of the shape features of the lower leg muscles

Shape features	DSA score ≤ 7<br>n = 36	DSA score > 7<br>n = 20	P value
Conventional features			
Actual volume (cm^3)	1084 ± 302	876 ± 268	0.01 *
Actual average area (cm^2)	32 ± 8	25 ± 7	0.004 *
Radiomic features			
Mesh volume	295894 ± 93221	271195 ± 96827	0.35
Voxel volume	295723 ± 92965	270611 ± 96630	0.34
Surface area	67341 ± 15050	82001 ± 26050	0.03 *
Surface area to volume ratio	0.26 ± 0.15	0.32 ± 0.12	0.10
Sphericity	0.32 ± 0.06	0.26 ± 0.06	<0.001 *
Maximum 3D diameter	145 ± 16	152 ± 26	0.19
Maximum 2D diameter slice	133 ± 16	129 ± 18	0.35
Maximum 2D diameter column	132 ± 17	127 ± 15	0.23
Maximum 2D diameter row	112 ± 13	121 ± 24	0.11
Major axis length	122 ± 15	121 ± 18	0.84
Minor axis length	82 ± 10	84 ± 13	0.66
Least axis length	57 ± 3	55 ± 7	0.54
Elongation	0.68 ± 0.09	0.70 ± 0.11	0.48
Flatness	0.47 ± 0.05	0.47 ± 0.07	0.89

Abbreviations: DSA = digital subtraction angiography.

Unless otherwise indicated, data are mean ± standard deviation.

* P values less than 0.05.

REFERENCES

[1] Stoner MC, Calligaro KD, Chaer RA et al (2016) Reporting standards of the Society for Vascular Surgery for endovascular treatment of chronic lower extremity peripheral artery disease. J Vasc Surg 64:e1-e21

[2] Stoner MC, Calligaro KD, Chaer RA et al (2016) Reporting standards of the Society for Vascular Surgery for endovascular treatment of chronic lower extremity peripheral artery disease: Executive summary. J Vasc Surg 64:227-228

[3] Rogers S, Carreira J, Phair A, Olech C, Ghosh J, McCollum C (2021) Comparison Between Below Knee Contrast Enhanced Tomographic 3D Ultrasound and CT, MR or Catheter Angiography for Peripheral Artery Imaging. Eur J Vasc Endovasc Surg 61:440-446

[4] DeCarlo C, Boitano LT, Schwartz SI et al (2021) Society for Vascular Surgery femoral runoff score is associated with limb-based patency after aortofemoral bypass. J Vasc Surg 74:124-133

[5] Elsharkawi M, Elsherif M, Ghoneim B et al (2021) Significance of Distal Runoff Score as a Key Influencer on Clinical Outcomes after Endovascular Interventions for Superficial Femoral Artery Disease. Ann Vasc Surg 73:234-243
